# Microbiome-metabolome analysis insight into the effects of high-salt diet on hemorheological functions in SD rats

**DOI:** 10.3389/fnut.2024.1408778

**Published:** 2024-09-24

**Authors:** Luming Qi, Yao Li, Zhixuan Chen, Changhong Wei, Xue Wen, Shuangyan Hu, Hang Wu, Zhuoheng Lv, Zhangmeng Xu, Lina Xia

**Affiliations:** ^1^School of Health Preservation and Rehabilitation, Chengdu University of Traditional Chinese Medicine, Chengdu, Sichuan, China; ^2^Jiangxi Province Key Laboratory of Traditional Chinese Medicine Pharmacology, Institute of Traditional Chinese Medicine Health Industry, China Academy of Chinese Medical Sciences, Nanchang, China; ^3^Department of Thoracic Surgery, National Cancer Center/National Clinical Research Center for Cancer/Cancer Hospital, Chinese Academy of Medical Sciences & Peking Union Medical College, Beijing, China; ^4^Department of Neck, Shoulder, Waist, and Leg Pain, Sichuan Province Orthopedic Hospital, Chengdu, Sichuan, China

**Keywords:** high-salt diet, hemorheological functions, microbiome-metabolome analysis, Mendelian randomization, SD rats

## Abstract

The present study examined the effect of two dietary regimens with elevated salt concentrations (4% and 8% salt) on hemorheological functions of SD rats, and explored the underlying mechanisms mainly through microbiome-metabolome analysis. An 8% HSD substantially altered the hemorheological parameters, and compromised intestinal barrier integrity and reduced the short-chain fatty acid levels. The microbiome-metabolome analysis revealed that 49 genus-specific microorganisms and 156 metabolites showed a consistent trend after exposure to both 4% and 8% HSDs. Pathway analysis identified significant alterations in key metabolites within bile acid and arachidonic acid metabolism pathways. A two-sample Mendelian randomization (MR) analysis verified the link between high dietary salt intake and hemorheology. It also suggested that some key microbes and metabolites (such as *Ruminococcaceae*_UCG-005, *Lachnospiraceae*_NK4A136, *Ruminiclostridium*_6, and *Ruminococcaceae*_UCG-010, TXB-2, 11,12-diHETrE, glycochenodeoxycholate) may involve in abnormalities in blood rheology caused by high salt intake. Collectively, our findings underscored the adverse effects of high dietary salt on hemorheological functions and provide new insight into the underlying mechanism based on microbiome-metabolome analysis.

## 1 Introduction

High-salt diet (HSD) is recognized as a significant determinant of cardiovascular diseases (1). Epidemiological studies consistently show that high sodium intake increases the risk of non-communicable diseases, especially hypertension, stroke, and other cardiovascular conditions. The underlying mechanisms through which HSD contributes to cardiovascular morbidity include the elevation of arterial pressure, vascular injury, and the promotion of inflammatory responses, among others. Reducing dietary salt is a cost-effective and practical strategy for preventing cardiovascular diseases (2). Aligning with this, the World Health Organization (WHO) recommends a daily salt consumption ceiling of 5 grams per individual. Nevertheless, high salt intake remains implicated in over 3 million fatalities globally each year, ranking as one of the top three diet-related hazards (3). A growing number of studies are being conducted to elucidate the negative effects of HSD.

The negative effects of an HSD on blood composition and flow properties could play a role in developing cardiovascular diseases. “The Yellow Emperor's Canon of Internal Medicine”, an ancient tome of Chinese medicine, notes that prolonged consumption of an HSD leads to increased viscosity and reduced flow rate. Contemporary studies corroborate these ancient findings, revealing that sustained HSD elevates sodium concentration, disrupts osmotic balance, and promotes water-sodium retention, resulting in endothelial dysfunction (1). Additionally, excessive salt intake is associated with augmented blood volume and inflammatory responses, precipitating the accrual of deleterious elements within the vasculature and consequently elevating blood viscosity (4, 5). Hemorheology, an interdisciplinary field at the intersection of biomechanics and biorheology, examines the macroscopic flow characteristics of blood, including cellular deformability and biochemical constituents (6). This expansive domain encompasses the study of blood flow, viscosity, deformation, and coagulation. Exploring hemorheological alterations may unveil novel insights into the cardiovascular diseases induced by an HSD.

As far as we know, cardiovascular compromise associated with an HSD extends beyond direct vascular effects, implicating multiple organs and systemic metabolic pathways. The intestinal milieu, a critical zone for salt absorption, experiences pronounced shifts in its ecosystem under HSD conditions, including alterations in the gut microbiota, intestinal barrier integrity, and epithelial cell function—factors that are increasingly recognized for their relevance to cardiovascular health (7). Historically, salt's antimicrobial properties have been harnessed for food preservation, but recent insights reveal that such high concentrations may disrupt gut microbial balance (8–10). These disruptions in the gut's ecological system, precipitated by a long-term HSD, may prove to be pivotal in cardiovascular damage. Notably, gut microbiota can synthesize or modify a plethora of metabolic products, including short-chain fatty acids (SCFAs). These metabolites, when translocated into the circulatory system, could perturb metabolic homeostasis and alter blood composition (11). It is postulated that a significant portion of mammalian blood metabolites—estimated to be at least 10%—are of gut microbiotal origin. These metabolites are implicated in the regulation of a spectrum of physiological and pathological processes, ranging from glucose, lipid, to amino acid metabolism (12, 13). Thus, the interconnections between gut microbiota, host metabolites, and hemorheological functions are becoming increasingly apparent. However, the tripartite interplay between gut microbiota, plasma metabolites, and hemorheology within the context of an HSD has not been thoroughly investigated.

In our current study, we established two dietary regimens with elevated salt concentrations (4% and 8% salt) to assess their effects on Sprague-Dawley (SD) rats over an 8-week period. We then examined the alterations in hemorheological functions utilizing an automated blood rheology analysis system. Concurrently, we evaluated the impact of these HSDs on the gut microbiome, the intestinal barrier, and the host metabolome, each assessed independently. The relationships among these parameters were further verified by a two-sample Mendelian randomization (MR) analysis. In summary, our study endeavored to contribute novel insights into the complex relationship between HSDs and cardiovascular health, opening avenues for further research.

## 2 Materials and methods

### 2.1 Animals and diet

Eighteen male Sprague-Dawley (SD) rats, aged 6 weeks, were procured from Sichuan Academy of Traditional Chinese Medicine (Sichuan, China). These animals were acclimatized under specific pathogen-free conditions with regulated temperature and humidity (50 ± 10% humidity; 12 h/12 h light cycle; 20.0 ± 2.0°C temperature) for 1 week. Subsequently, the rats were randomly divided into three groups (*n* = 6), each subjected to a distinct diet: a normal salt diet (NSD) containing 0.5% NaCl, and HSDs with 4% and 8% NaCl, respectively, which are the most commonly used dietary patterns in the published studies (9, 14). The diets, formulated to meet the nutritional needs of growing rats, were supplied by SiPeiFu Biotechnology Co., Ltd. (Beijing, China). Unlimited access to food and water was ensured throughout the study. After 8 weeks, each rat was placed in the anesthesia chamber connected to the gas anesthesia machine (RWD Life Science Co., R500, Shenzhen, China) and turned on the isoflurane vaporizer with an initial concentration of 3.5% to induce anesthesia. Once the rat was fully anesthetized, adjusted the maintenance concentration to ~1.5%, then removed the rat from the anesthesia chamber and placed it on the dissection table equipped with an anesthesia mask. After collecting the target tissues, euthanize the rat by exsanguination. This procedure ensures that the rat remains in a state of deep anesthesia and is completely pain-free. The isoflurane was purchased from RWD Life Science Co. (Shenzhen, China; Batch No. 21110601). This animal experiment study complies with relevant ethical guidelines and international standards. The studies involving animals were reviewed and approved by the Experimental Animal Welfare and Ethics Committee of Chengdu University of Traditional Chinese Medicine (Affiliation: Chengdu University of Traditional Chinese Medicine; Protocol number: 2018-21). For transparency, we emphasize that the ethical approval number 2020-36 used in the previous version was incorrect. We have now changed it to the correct ethical approval number 2018-21.

### 2.2 Hemorheology test

Hemorheological assessments were conducted within 4 h post blood collection (15). Approximately 5 mL of blood was drawn into a heparin sodium anticoagulant tube for whole blood viscosity (WBV) and plasma viscosity (PV) measurements, while about 2 mL was reserved in a closed tube for erythrocyte sedimentation rate (ESR) analysis. WBV and PV determinations were facilitated using an automated blood rheology analyzer (SA-9000, Beijing Succeeder Technology Inc., China). For ESR and hematocrit measurements, around 1.6 mL of whole blood was collected in a tube containing 3.8% sodium citrate, analyzed using an SD-100 dynamic hematocrit tester (Beijing Succeeder Technology Inc., China). Indices such as erythrocyte deformation (ED), erythrocyte rigidity (ER), erythrocyte aggregation (EA), and reduced blood viscosity (RBV) at low and high shear rates (LSR and HSR) were calculated using standard formulas.

### 2.3 Histological and immunofluorescence analysis

Aortic and colonic tissues were fixed in 4% paraformaldehyde for over 24 h, dehydrated in graded alcohols, and embedded in paraffin to produce 5 μm sections for hematoxylin and eosin (H&E) staining. Immunofluorescence staining utilized primary antibodies against ZO-1, occludin, claudin-1, and claudin-2, followed by incubation with FITC-labeled secondary antibodies. Nuclear counterstaining was performed with 4′,6-diamidino-2-phenylindole (DAPI) (Servicebio, Wuhan, China). Imaging was conducted using an Olympus Confocal Microscope.

### 2.4 SCFAs determination

Short-chain fatty acids (SCFAs) in feces were extracted and analyzed with modifications to previously established methods (16). Before a final centrifugation, fecal samples were homogenized in ultrapure water to create a 20% slurry, centrifuged, and the supernatant acidified with 25% metaphosphoric acid to reduce the loss of SCFAs. The supernatant was analyzed using an Agilent 7820A gas chromatograph (Agilent Technologies, Inc., USA) with a DB-FFAP column, following a specific temperature program. SCFA concentrations were quantified using the standard curve method.

### 2.5 16s rRNA sequencing

Total genomic DNA was extracted from fecal samples using a stool DNA kit (Cwbio, Jiangsu, China), with DNA quality and concentration assessed via agarose gel electrophoresis and NanoDrop^®^ ND-2000 spectrophotometer (Thermo Scientific Inc., USA). Amplification of the V3-V4 region of the 16S rRNA gene utilized universal primers, with PCR products purified and quantified before sequencing on the Illumina NovaSeq 6000 platform. Data processing was performed using the QIIME2 pipeline, with sequences assigned to operational taxonomic units (OTUs) via DADA2 and taxonomic analysis conducted with the VSEARCH algorithm against the Silva database (17).

### 2.6 Host metabolome determination

100 μL of plasma was vortexed with 200 μL of acetonitrile for 30 seconds, centrifuged, and the supernatant dried and reconstituted in 200 μL acetonitrile: water (4:1). After vortexing and centrifugation, the clear supernatant was filtered for analysis. Quality control (QC) samples were inserted at 3-needle intervals to ensure method reliability. Plasma metabolites were analyzed using an ultra-high performance liquid chromatography coupled with hybrid quadrupole-orbitrap high-resolution mass spectrometry (UHPLC/orbitrap-MS) method based on an ACQUITY UPLC HSS T3 column (2.1 mm × 100 mm, 1.8 μm). The mobile phase A is 99% water with 0.1% formic acid, and the mobile phase B is 99% acetonitrile with 0.1% formic acid. The gradient ranged from 1% to 99% B over 27 minutes, with an injection volume of 2 μL and a flow rate of 0.3 mL/min. The MS acquisition was conducted on both positive and negative ionization modes. The heated electrospray ionization parameters were set as follows: sheath gas flow 55 arb, auxiliary gas flow 15 arb, spray voltage 3.5 kV for positive ionization and 3.8 kV for negative ionization, capillary temperature 350°C, and probe heater temperature 300°C (17).

The raw data were imported into the metabolomics processing software Progenesis QI (Waters Corporation. Milford, USA) for baseline filtering, peak identification, integration, retention time correction, etc., and finally obtained a data matrix containing retention time, mass-to-charge ratio and peak intensity information. The software was then used to search the library for the identification of characteristic peaks, match the MS and MS/MS mass spectral information with the metabolic databases. The MS mass error was set to < 10 ppm, and the annotation of metabolites was based on the secondary mass spectral matching scores. The main databases include mainstream public databases such as “LIPID MAPS” (https://lipidmaps.org/), “HMDB” (https://hmdb.ca/), and “METLIN” (https://metlin.scripps.edu/), and the self-built databases (Supplementary Figure S1).

### 2.7 MR analysis

To further confirm the experimental results, we employed a two-sample MR approach to explore potential causal links, leveraging large-scale GWAS datasets to mitigate confounding factors (18). Notably, due to the unavailability of certain genetic variants, we selectively validated associations using available single nucleotide polymorphisms (SNPs) or analogous phenotypes. We sourced SNPs indicative of salt intake exposure from GWAS data on a European cohort of 396,020 individuals from the UK Biobank (19). For hemorheological characteristics, GWAS data on seven red blood cell phenotypes and one fibrinogen phenotype were obtained from the IEU database, serving as proxies for the indices reflective of hemorheological traits in this study (20, 21). These indicators including red blood cell count (RBC), red cell distribution width (RDW), mean corpuscular volume (MCV), hematocrit, hemoglobin concentration (HGB), mean corpuscular hemoglobin (MCH), and mean corpuscular hemoglobin concentration (MCHC) are also closely associated with the rheological properties of blood. SNPs pertaining to gut microbiota and blood metabolites were extracted from the MiBioGen consortium and GWAS Catalog database datasets, respectively (22–26). Detailed phenotype information is listed in Supplementary Table S1.

We applied four analytical methods: inverse variance weighted (IVW), weighted median (WM), MR-Egger (ME), and Simple mode (SM), with IVW as the primary method (24). Single nucleotide polymorphisms (SNPs) associated with outcomes were selectively excluded based on a significance threshold of *P* < 5 × 10^−8^ for the salt intake phenotype and *P* < 1 × 10^−5^ for the phenotypes related to gut microbiota and blood metabolites, and the F-statistic was used to counteract weak instrumental variables. This parameter selection was informed by methodologies outlined in our prior publication (27).

### 2.8 Other data analysis

The measurements were presented as mean ± SD. Group differences were assessed using Fisher's LSD test via SPSS (Version 13.0, SPSS Inc., Chicago, USA). Microbial community richness and diversity were quantified using Chao1, ACE, Shannon, and Simpson indices. Microbiome variation was depicted through principal coordinate analysis (PCoA), permanova analysis and clustering heatmap. For the plasma metabolome, principal component analysis (PCA) and partial least squares discriminant analysis (PLS-DA) were used to visualize the variation among different groups. MBROLE 2.0 (http://csbg.cnb.csic.es/mbrole2/analysis.php) was used to annotate the biological pathways of differential metabolites. Volcano plot was used to identify the differential microbes/metabolites according to P < 0.05 and |FC|>1.2. The *P* value was adjusted by the false discovery rate (FDR) method. Spearman's rank correlation was applied to analyze the correlation of differential features derived from hemorheology, gut microbiome and plasma metabolome. “MetOrigin” (https://metorigin.met-bioinformatics.cn/home/) was applied to identify microbiota related metabolites and “Gephi” (0.10.1 version) software was used to present the network association (28).

## 3 Results

### 3.1 HSD affects general characteristics and hemorheological functions of SD rats

**Figure 1:**
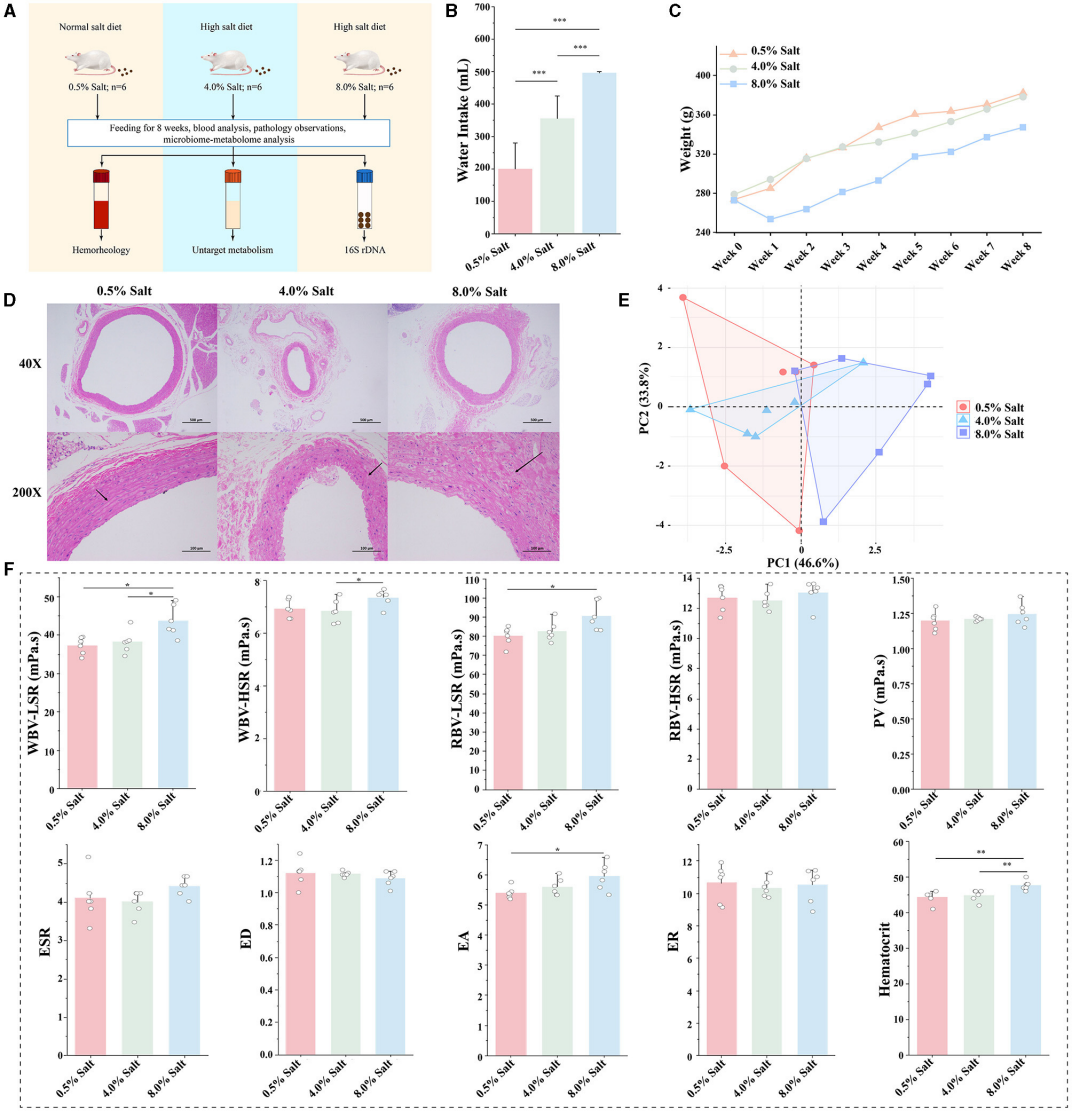
HSD affects general characteristics and hemorheological functions of SD rats. **(A)** Experimental design; **(B)** the change of drinking water; **(C)** the change of body weight; **(D)** H&E staining of the aorta; **(E)** the overall effect of HSDs on hemorheological functions; **(F)** the effect of the HSD on each parameter of hemorheology. *p < 0.05; **p < 0.01; ***p < 0.001.

The study design is depicted in Figure 1A. During the feeding period, rats in the HSD groups consumed more water to maintain osmotic pressure balance within their bodies (Figure 1B). By the conclusion of the 8-week HSD period, substantial changes in the average body weight of SD rats across the different groups were observed. Specifically, the 8% NaCl HSD cohort demonstrated an abrupt weight decrease initially, indicating potential difficulties in adapting to the high-salt conditions (Figure 1C).

H&E staining revealed no significant histopathological alterations in the aortas of the NSD group (Figure 1D). Conversely, the aortas from the 8% NaCl HSD displayed structural deformities in the vascular wall, endothelial damage, and localized inflammatory cell infiltration. Similar, albeit milder, observations were made in the 4% NaCl HSD group. These findings suggest that prolonged high dietary salt intake may lead to detrimental changes in aortic vessel morphology and integrity.

To assess hemorheological functions, we constructed a two-dimensional plot based on PCA, highlighting discernible differences among the groups. In Figure 1E, the HSDs affected the hemorheological characteristics of SD rats, particularly within the 8% NaCl group, which diverged markedly from the samples in NSD. The 8% NaCl diet significantly increased the parameters of WBV-LSR and RBV-LSR (*P* < 0.05). The 8% salt HSD also significantly increased the parameter of EA and hematocrit (*P* < 0.05). In addition, 8% salt HSD mildly increased the levels of WBV-HSR, RBV-HSR, PV and ESR. Conversely, the 4% NaCl HSD did not exhibit significant changes (Figure 1F). These findings suggested that high concentration salt intake affected hemorheological functions, primarily resulting in increased blood viscosity in SD rats.

### 3.2 HSD impairs barrier integrity and reduces SCFA levels in the gut of SD rats

**Figure 2:**
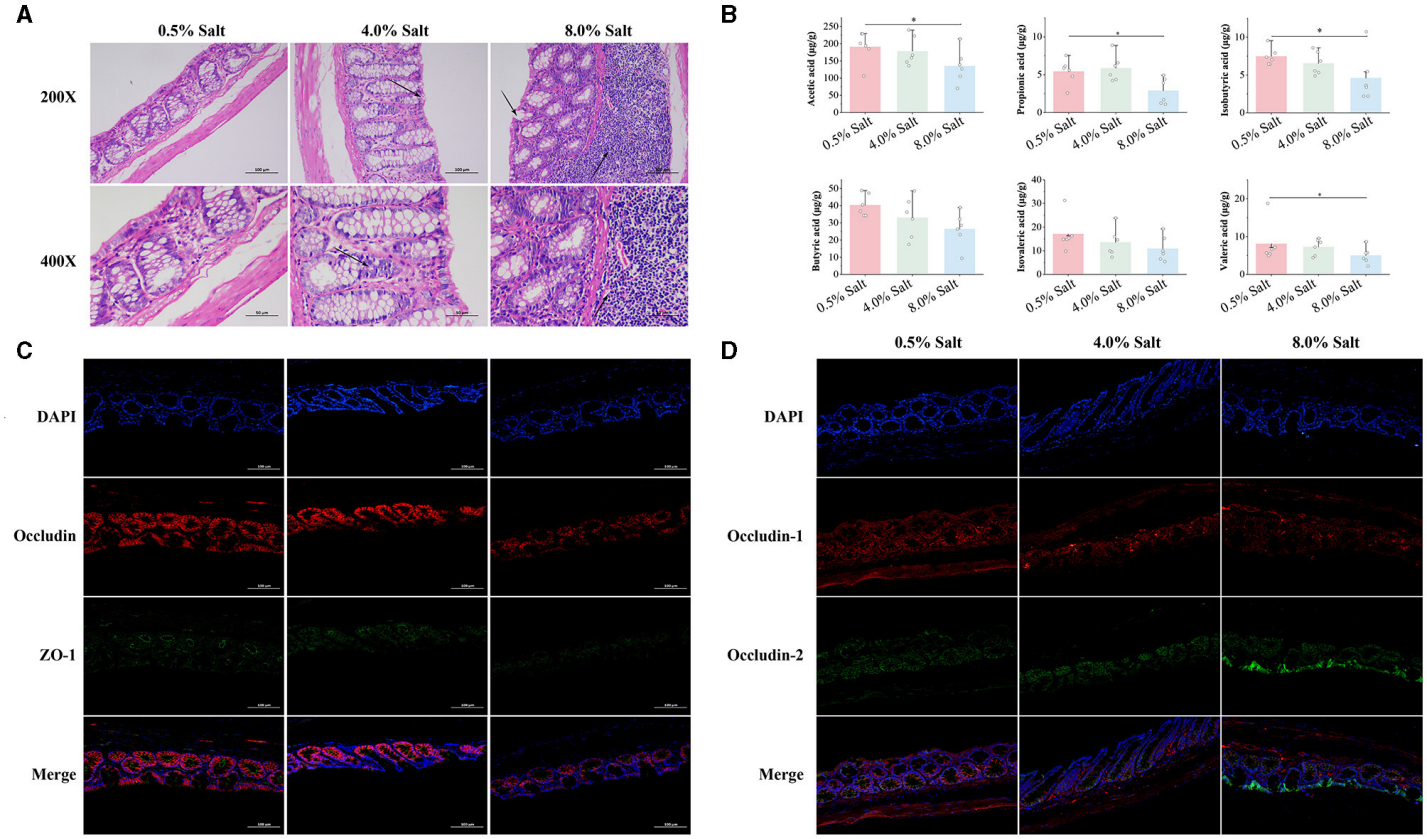
HSD impairs barrier integrity and reduces SCFA levels in the gut of SD rats. **(A)** The histological examination of colonic sections; **(B)** the expression of tight junction proteins ZO-1 and Occludin; **(C)** the expression of tight junction proteins Claudin-1 and Claudin-2; **(D)** the levels of SCFAs in the gut. *p < 0.05.

Intestinal barrier function plays a crucial role in controlling the translocation of gut components into the bloodstream, thereby affecting overall blood health. This study examined the impacts of 4% and 8% salt diets on the intestinal morphology and integrity in SD rats. Histological examination of colonic sections revealed that the normal salt diet preserved intestinal morphology and epithelial integrity, while higher salt concentrations led to noticeable disruptions. Notably, an 8% salt diet resulted in substantial mucosal integrity loss and significant inflammatory infiltration (Figure 2A). Moreover, we observed a salt-dependent decline in the expression of crucial tight junction proteins ZO-1, Occludin, and Claudin-1, which were indicative of increased intestinal permeability (Figures 2C, D).

SCFAs, synthesized by gut microbiota, play a vital role in maintaining intestinal barrier function and overall gut health by protecting the intestinal mucosa, providing nutrients to epithelial cells, and altering intestinal PH. We quantified six SCFAs across SD rat groups subjected to varying salt concentrations in their diets. HSD, particularly the 8% salt regimen, significantly depleted SCFA levels, notably propionic, isobutyric, butyric acids, and isovaleric acid in the feces (Figure 2B). This evidence were indicative of the adverse effects of high-salt consumption on intestinal morphology, barrier integrity, and SCFA production, establishing a clear link to dietary salt levels.

### 3.3 HSD reshapes the gut microbiota profiles and alters specific microbial taxa in SD rats

**Figure 3:**
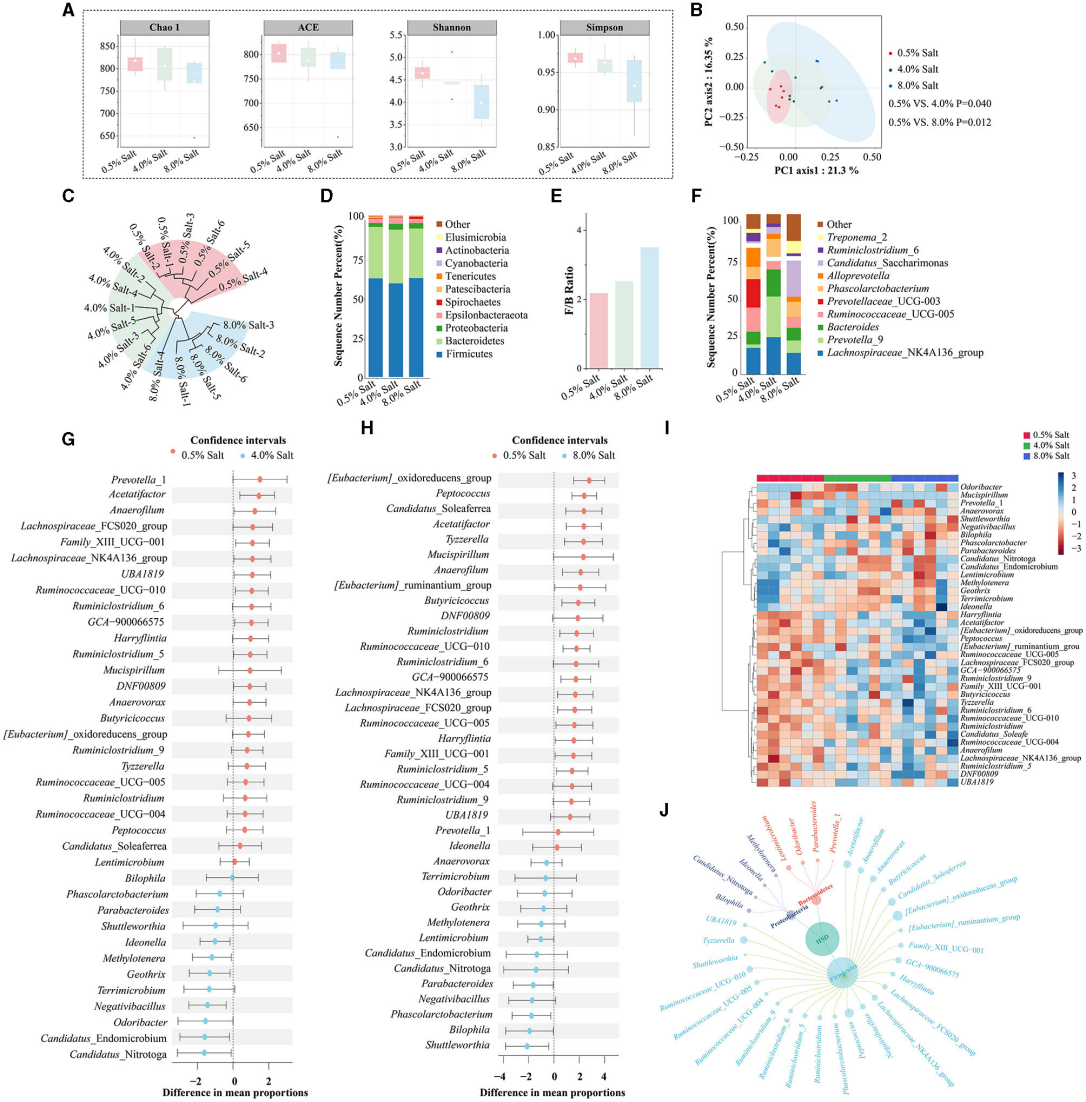
HSD reshapes the gut microbiota profiles and alters specific microbial taxa in SD rats. **(A)** The α-diversity analysis; **(B)** PCoA analysis; **(C)** tree clustering diagram; **(D)** the abundance of gut microbiota at the phyla level; **(E)** Firmicutes/Bacteroidetes ratio; **(F)** the abundance of gut microbiota at the genus level; **(G)** the differential bacterial genera after 4% salt diet; **(H)** the differential bacterial genera after 8% salt diet; **(I)** the cluster heatmap analysis based on differential bacterial genera; **(J)** the petal diagram of the main differential bacteria.

To elucidate the effects of an HSD on gut microbiota, we conducted 16S rRNA gene sequencing on fecal samples from SD rats fed diets with different salt concentrations. The sequencing yielded over 1.4 million high-quality reads, averaging nearly 80,000 reads per sample, and identified over a million OTUs. The α-diversity analysis showed a decrease in gut microbiota diversity in SD rats fed 4% and 8% salt diets, indicated by lower Shannon and Simpson indices (Figure 3A). The β-diversity analysis utilized PCOA to delineate the distinct microbial phenotypes across groups at the OTU level. This analysis demonstrated a significant separation of samples correlated with different dietary salt concentrations (Figure 3B). Additionally, PERMANOVA supported these findings, showing substantial variations in gut microbiota profiles among the groups, which was further corroborated by tree clustering diagram (Figure 3C).

After filtering for low-abundance bacteria and normalizing data, taxonomic annotation identified a diverse microbial presence across the samples, encompassing 19 phyla, 78 families, and 165 genera. The gut microbiota was predominantly composed of Firmicutes (60.10%) and Bacteroidetes (31.95%) (Figure 3D). HSDs obviously increasing the phyla Actinobacteria and Verrucomicrobia, while decreasing the phyla Spirochaetes and Patescibacteria. The Firmicutes/Bacteroidetes ratio, an indicator of gut microbiota balance, shifted toward dysbiosis in rats fed with 8% salt, underscoring the impactful role of diet on gut microbial ecology (Figure 3E).

At the genus level, our analysis revealed significant shifts in the gut microbiota composition of SD rats on 4% and 8% salt diets. Core bacterial genera such as *Lactobacillus* (11.86%), *Ruminococcaceae*_UCG-014 (7.94%), *Eubacterium_coprostanoligenes*_group (6.38%), and *Lachnospiraceae_NK4A136_group* (6.03%) were among the most abundant (Figure 3F). The study observed a downregulation in the relative abundance of 12 bacterial genera and an upregulation of 8 others post-4% salt diet (Figure 3G, Supplementary Table S2). An 8% salt diet further altered the bacterial profile, downregulating 22 genera and upregulating 5 others (Figure 3H, Supplementary Table S2). A total alteration pattern was noted in 38 bacterial genera across both salt diet conditions. Cluster heatmap analysis effectively distinguished rats based on dietary salt concentration through shifts in bacterial genera abundances (Figure 3I). The variations in gut microbiota induced by HSDs were predominantly observed in the Firmicutes, Bacteroidetes, and Proteobacteria phyla (Figure 3J). This suggested that prolonged high-salt consumption dramatically shifted specific microbial taxa, disrupting gut microbiota balance in SD rats.

### 3.4 HSD alters host metabolic levels and highlighted bile acid and arachidonic acid metabolism

**Figure 4:**
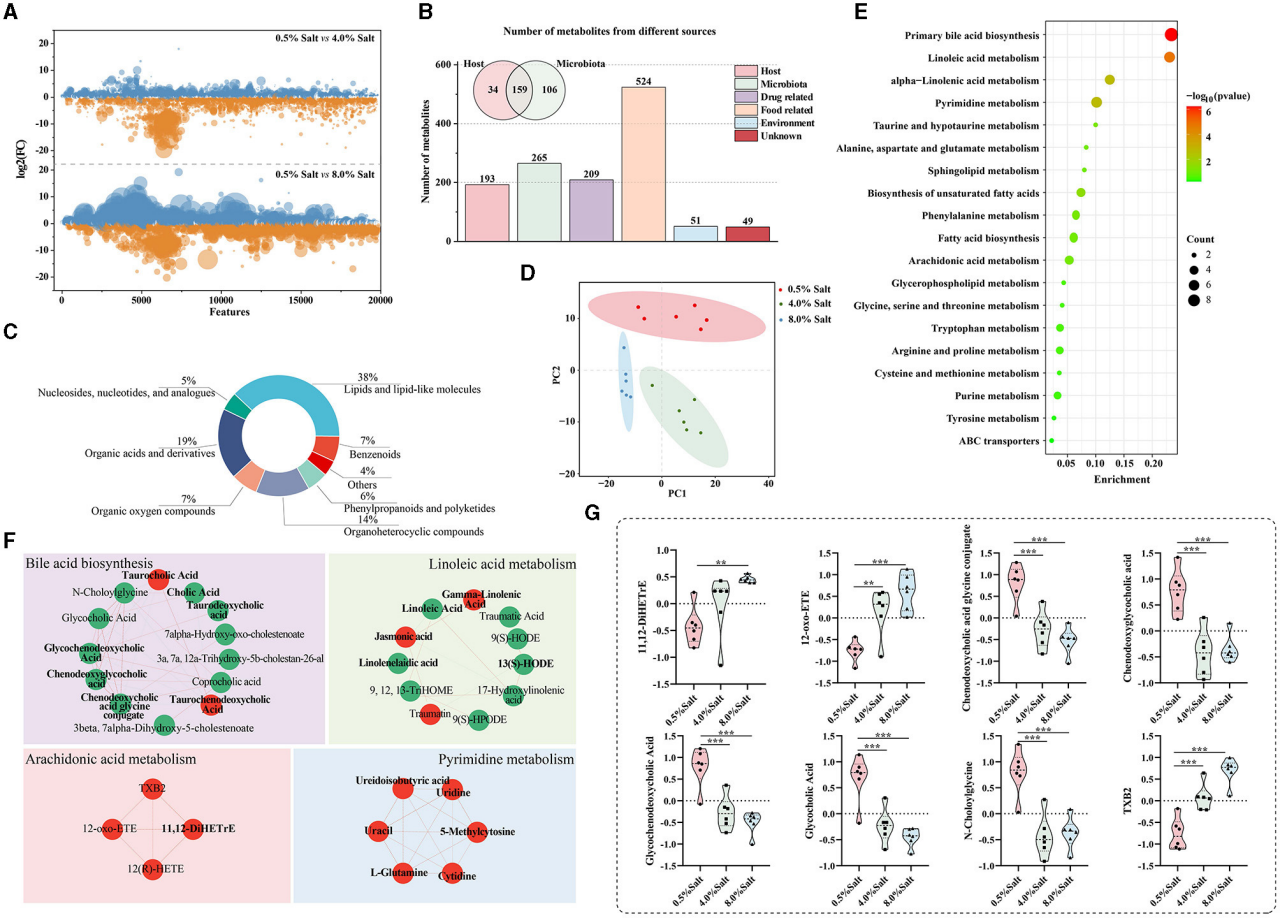
HSD alters plasma metabolic levels and highlighted bile acid and arachidonic acid metabolism. **(A)** The disruptions in the ionic fragmentation profiles after the HSDs; **(B)** the origins of metabolites based on the “MetOrigin” tool; **(C)** the categories of the metabolites; **(D)** the result of the PLS-DA model; **(E)** the annotation of biological pathways and microbiota-related compounds in bold; **(F)** the differential metabolits in the key biological pathwas; **(G)** the abundance of metabolites in arachidonic acid and bile acid metabolic pathways. *p < 0.05; **p < 0.01; ***p < 0.001.

HSD-induced modifications in the gut microbiota can systematically affect host metabolism and blood properties. To investigate the effects of HSDs on blood metabolomics, we utilized UHPLC-Q Exactive HF-X for untargeted metabolomic profiling to analyze plasma metabolite composition. A total of 19,865 precursor m/z values were detected, respectively, in both positive and negative ion modes. We ensured data quality by retaining features with relative standard deviations (RSD) < 30%, and PCA of QC samples confirmed the method's reliability (Supplementary Figures S2, S3). Our findings indicated that 8-week HSDs significantly altered SD rats' metabolites, with 8% salt diets causing notably greater disruptions in the ionic fragmentation profiles (Figure 4A).

A total of 636 plasma metabolites retrieved from KEGG database were retained to further analyze. To deepen our understanding of the gut microbiota's influence, we employed the “MetOrigin” tool for the selective identification of microbiota-related compounds. This analysis revealed that a significant portion of metabolites, 265 in total, were linked to microbiota, with 159 resulting from co-metabolism with the host and 106 originating directly from the gut microbiota (Figure 4B). These metabolites predominantly belonged to categories of lipids and lipid-like molecules (38.1%), organic acids and derivatives (18.9%), and organoheterocyclic compounds (14.3%) (Figure 4C). These data supported the crucial role of the gut microbiota in regulating host metabolism.

PLS-DA models revealed distinct clustering patterns corresponding to varied diets in SD rats (Figure 4D). After exposure to an HSD containing 4% salt, the relative abundances of 47 metabolites were significantly downregulated, while 48 metabolites exhibited marked upregulation (Supplementary Table S3). The HSD with 8% salt led to substantial downregulation of 72 metabolites and significant upregulation of 84 metabolites (Supplementary Table S3). Remarkably, 156 compounds (77.14%) among these differentially expressed metabolites displayed a consistent trend following exposure to both 4% and 8% HSD.

To provide biological pathway context, we conducted enrichment analysis of categorical annotations using the “MBROLE” database. Five microbiota-related metabolic pathways, including “Primary bile acid biosynthesis,” “Linoleic acid metabolism,” “Pyrimidine metabolism,” “alpha-Linolenic acid metabolism,” “Biosynthesis of unsaturated fatty acids,” and “arachidonic acid metabolism,” exhibited significant alterations following HSD feeding (Figure 4E). Notably, after 8 weeks of high salt intake, a widespread reduction was observed in the levels of metabolites related to bile acid and linoleic acid metabolism, alongside an elevation in pyrimidine and arachidonic acid metabolites. These significantly altered metabolic pathways are all related or partially related to gut microbial metabolism (the microbiota-related compounds presented in bold front) (Figure 4F). Additionally, we meticulously focused on the top 20 significantly altered metabolites, unveiling that three and five of them, respectively, were associated with the arachidonic acid metabolic and bile acid metabolic pathways (Figure 4G). These results demonstrated that HSDs with different salt levels dramatically affect the host metabolite profile and microbiota-associated metabolic pathways, especially arachidonic acid and bile acid metabolic pathways.

### 3.5 The MR analysis verified for the association of hemorheological abnormality to gut microbiota and plasma metabolites after HSDs

**Figure 5:**
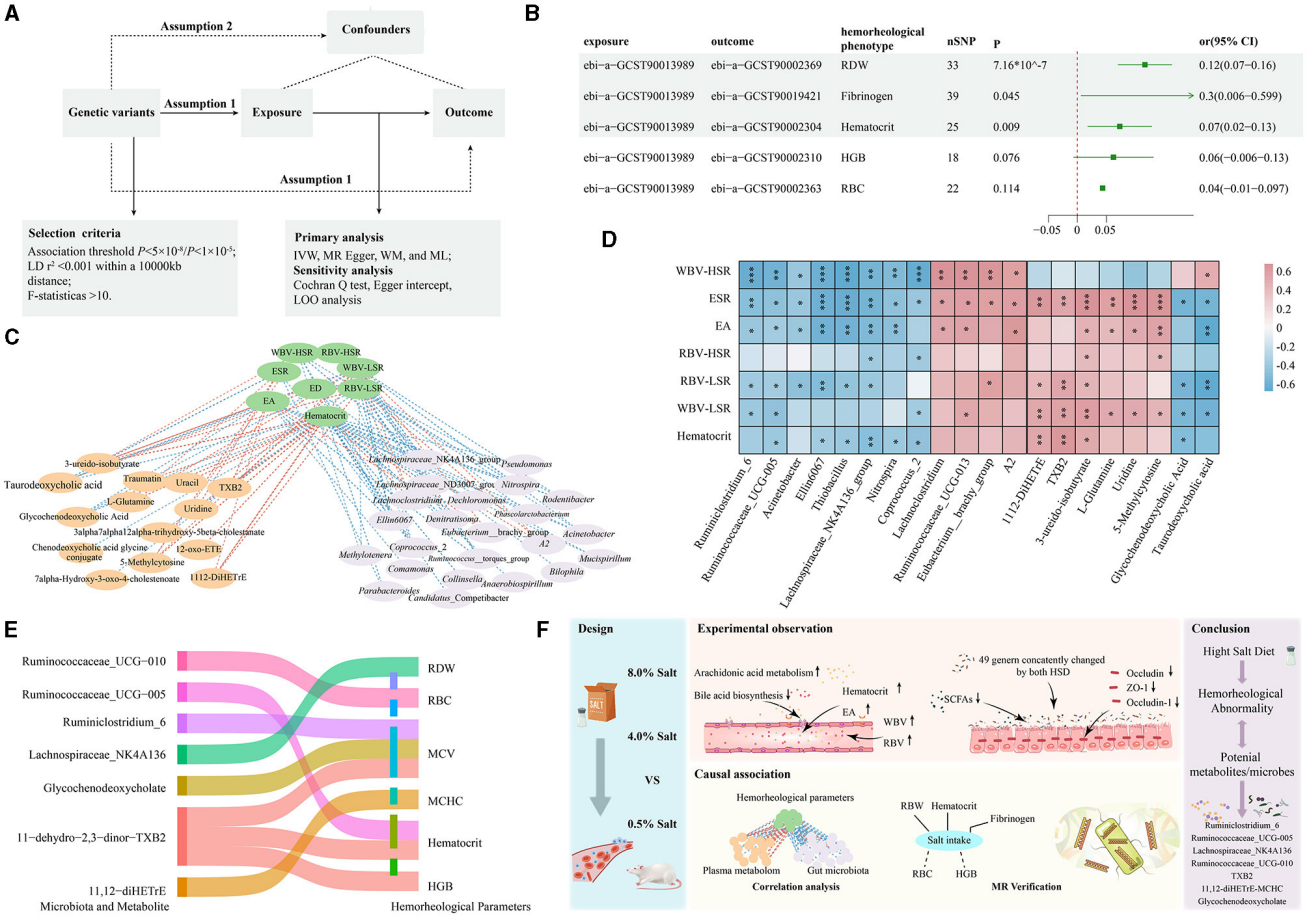
MR analysis strengthened the molecular evidence for the association of hemorheological abnormality to gut microbiota and plasma metabolites after HSDs. **(A)** The parameters of MR analysis; **(B)** the MR results between high dietary salt intake and the selected hemorheological phenotypes; **(C)** the results of Spearman's rank correlation analysis; **(D)** the correlation between the key bacteria genus, key metabolites and hemorheological parameters; **(E)** the causal relationships of associated gut microbiota and plasma metabolites with the selected hemorheological phenotypes; **(F)** the overview diagram of this study. *p < 0.05; **p < 0.01; ***p < 0.001.

Considering the genetic influence on hemorheology, as well as on microbiome and metabolome characteristics, we employed a two-sample MR to verify the connection between high dietary salt intake and hemorheological changes, and emphasized the potential mediating roles of gut microbiota and related metabolites at the genetic level (Figure 5A). Following a rigorous MR screening process, 47 SNPs representing sodium intake were retained as instrumental variables. For hemorheological-related outcomes, the IVW test revealed significant positive correlations between salt intake and RDW (beta = 0.116; *P* = 7.16 × 10^−7^), hematocrit (beta = 0.073; *P* = 0.009), and fibrinogen (beta = 0.303; *P* = 0.045) (Figure 5B). Additionally, RBC and HGB parameters were positively correlated with salt intake. This conclusion from MR analysis further verify the association between high dietary salt intake and hemorheological abnormality.

Based on the results of randomized controlled trials, the cross-correlation-based network based on Spearman's rank correlation analysis revealed that 87 features from the gut microbiome and 39 features from the plasma metabolome were significantly associated with hemorheological parameters (Figure 5C). Notably, certain beneficial bacteria genera (e.g., *Lachnospiraceae*_NK4A136_group, *Ruminiclostridium*_6, *Ruminococcaceae*_UCG-005, *Ruminococcaceae*_UCG-010) were negatively correlated with blood viscosity, while harmful bacteria genera (e.g., *Lachnoclostridium, Eubacterium*_brachy_group) showed positive correlations with various hemorheological parameters. For the plasma metabolites, compounds like 11,12-DiHETrE and TXB2 from arachidonic acid metabolism, and 3-ureido-isobutyrate, 5-Methylcytosine, and uridine from pyrimidine metabolism showed significant positive correlations with various hemorheological parameters. Conversely, glycochenodeoxycholate and taurodeoxycholate levels from bile acid metabolism were significantly inversely correlated with hemorheological measures (Figure 5D).

Next, we used MR analysis to verify these correlations from randomized controlled trials. 23 gut microbiota and 19 host metabolite phenotypes with available genetic variations were screened. Surprisingly, significant negative causal correlations were found between Ruminiclostridium_6 and MCV (beta = −0.034; *P* = 0.0003), Ruminococcaceae_UCG-005 and Hematocrit (beta = −0.02015; *P* = 0.037), Lachnospiraceae_NK4A136 and RDW (beta = −0.0172; *P* = 0.038), and Ruminococcaceae_UCG-010 and RBC (beta = −0.030; *P* = 0.039). For the metabolites, 11-dehydro-2,3-dinor-TXB2 presented significant positive causal correlations with MCV (beta = 0.011; *P* = 0.039), HGB (beta = 0.015; *P* = 0.004) and Hematocrit (beta = 0.017; *P* = 6.78e-05). Additionally, 11,12-diHETrE exhibited significant positive causal correlations with MCHC (beta = 0.012; *P* = 0.006), while glycochenodeoxycholate levels showed significant negative causal correlations with MCV (beta = −0.011; *P* = 0.028) (Figure 5E). The detail of MR results was provided in Supplementary Table S4. These findings confirm that certain key microbes (such as Ruminiclostridium_6, Ruminococcaceae_UCG-005, Lachnospiraceae_NK4A136, Ruminococcaceae_UCG-010) and metabolites (such as TXB2, TXB2, glycochenodeoxycholate) may play an important role in the process of hemorheological abnormality caused by an HSD.

## 4 Discussion

HSD is a significant risk factor for cardiovascular diseases, with its underlying mechanisms being complex. Research on the association between HSD and the cardiovascular system primarily focuses on diseases such as hypertension, stroke and heart failure (8–10, 29). As an important early warning indicator for cardiovascular diseases, few studies have explored the impact of a long-term HSD on the hemorheological characteristics of blood. In this study, we established two HSD pattern containing 4% and 8% sodium chloride to investigate the effects of different salt diets on the rheological characteristics of SD rats' blood and explored potential associative mechanisms based on gut microbiota and plasma metabolomics.

Hemorheology is the science that applies principles of physics to analyze the characteristics of blood flow within blood vessels. The primary parameters it generates reflect the physical and hydrodynamic properties of blood during flow, holding significant clinical relevance in the prevention and treatment of cardiovascular diseases (30). Ancient Chinese medical texts, such as the “The Yellow Emperor's Canon of Internal Medicine”, note that a long-term HSD affects blood circulation. Our results indicated that an 8% NaCl HSD significantly increased blood viscosity and coagulability, confirming the scientific basis of ancient medical theories. Changes in hemorheological functions may be a key factor in the predisposition to cardiovascular diseases caused by HSD.

The gut microbiota has been proven to be a significant factor affecting cardiovascular health (31). Since the gut is the primary site for food absorption, changes in diet will inevitably disrupt the original balance of the gut ecosystem. We hypothesized that the changes in the rheological characteristics of SD rats' blood due to an HSD may be related to the gut barrier, gut microbiota, and their associated metabolites. The research results confirmed the comprehensive impact of the HSDs on the gut ecosystem, including the reduction of intestinal tight junction proteins and short-chain fatty acid content, and causing dysbiosis of gut microbiota. Additionally, we found that these changes were correlated with the salt concentration in the diet, which was, the higher the salt content in the diet, the greater the damage to the gut ecosystem. These findings greatly enrich previous research results (32, 33).

Host metabolism is also key in influencing blood rheological functions. We utilized UPLC-Q-Orbitrap HRMS technology to study the impact of different concentrations of HSDs on the plasma metabolome. This technology offers exceptional performance for the capture and identification of non-target metabolites. We found that HSDs significantly altered the abundance of various plasma metabolites, mainly focusing on pathways such as bile acid metabolism, arachidonic acid, and pyrimidine metabolism. The association between bile acid metabolism and blood rheological properties is reflected in the digestion and absorption of fats and inflammatory responses (34, 35). Abnormalities in bile acid metabolism may indirectly affect the fluidity and viscosity of blood by influencing cholesterol metabolism and increasing the concentration of inflammatory cells and proteins in the blood. Arachidonic acid metabolism is closely related to the contraction and relaxation of blood vessels. Arachidonic acid can be converted into components such as 11,12-DiHETrE (Molecular weight: 338.4816) and 12-oxo-ETE (Molecular weight: 318.4504) through cytochrome P450 epoxygenase (34, 36). These compounds can stimulate potassium ion channels, producing vasodilatory effects. TXB2 (Molecular weight: 370.4804) is a key metabolic product in the arachidonic acid metabolism pathway. Its increased content signifies platelet aggregation and vascular contraction, playing a significant role in blood coagulation and vascular reactivity. Notably, the majority of the differential metabolites are related to microbiota, indicating that the gut microbiota-metabolism axis is an important mechanism by which HSD induces abnormal blood rheology.

To further confirm the impact of an HSD on hemorheological functions and verify whether gut microbiota and host metabolism are involved in this process, we innovatively applied the two-sample MR technique to explore the associations at the genetic level. Compared to randomized controlled trials, this method can overcome issues such as high implementation requirements, strict control, and difficulty, while also saving time in experimentation. We observed the causal relationships of SNPs representing salt intake with hematocrit, RDW, and fibrinogen. The parameters are closely related to the hemorheological function, greatly enhancing the reliability of the randomized controlled experimental results. Through MR validation, we have identified some key pairs from the gut microbiome and plasma metabolome that are involved in the process of hemorheological abnormality caused by high salt intake. They are Ruminiclostridium_6-MCV, Ruminococcaceae_UCG-005 -Hematocrit, Lachnospiraceae_NK4A136-RDW, Ruminococcaceae_UCG-010-RBC, TXB2-MCV, TXB2-HGB, TXB2-Hematocrit, 11,12-diHETrE-MCHC, and glycochenodeoxycholate (Molecular weight: 449.6233)-MCV. These pairs are very valuable for further understanding the molecular mechanisms behind the hemorheological abnormality caused by high salt intake. The overall article overview diagram is shown in Figure 5F.

Finally, this study has the following limitations. Firstly, due to the lack of available SNPs for hemorheological parameters obtained the automated blood rheology analyzer, we could only select several similar phenotypes for MR analysis in this study to clarify the impact of an HSD on hemorheological functions. Secondly, the specific microbiota and plasma metabolites involved in the abnormal blood rheology caused by an HSD require further in-depth research by setting up additional randomized controlled trials. Thirdly, appropriate internal standards should be used and correctly added to the sample, which can improve the accuracy and reliability of metabolite measurements.

## 5 Conclusion

In summary, this study has observed the adverse effects of an HSD on hemorheological function and explored the underlying mechanisms through microbiome-metabolome analysis. With MR validation, we identified some key microbes and metabolites involved in abnormalities in blood rheology caused by high salt intake. This study can provide new insights into the association between the HSD and cardiovascular diseases.

## Data Availability

The original contributions presented in the study are publicly available. This data can be found here: https://www.ncbi.nlm.nih.gov/bioproject/1128423/PRJNA1128423.
