# Supplementary material for: Microbiome-metabolome analysis insight into the effects of high-salt diet on hemorheological functions in SD rats
Source: Front Nutr. 2024 Sep 24;11:1408778. doi: 10.3389/fnut.2024.1408778 (PMC11460366; doi:10.3389/fnut.2024.1408778)
Supplement: Supplementary file 1 [file Image_1.pdf]

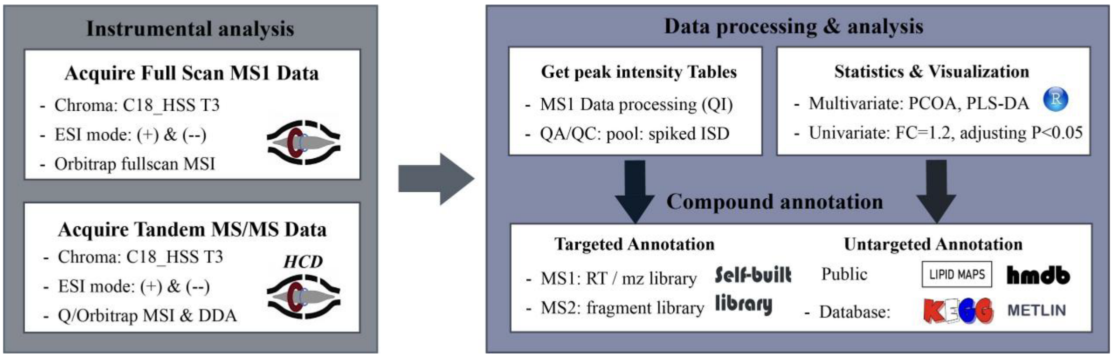

Figure S1: Non-targeted metabolomic analysis and data processing flow

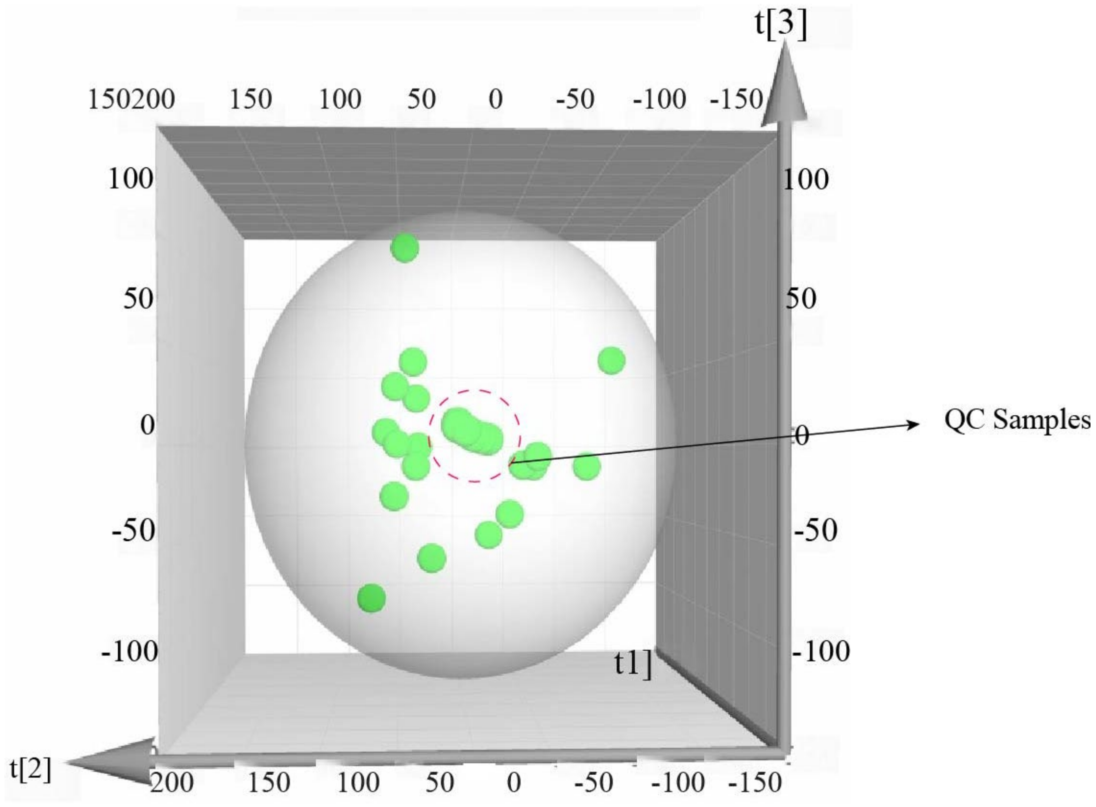

Figure S2: The PCA results of QC samples

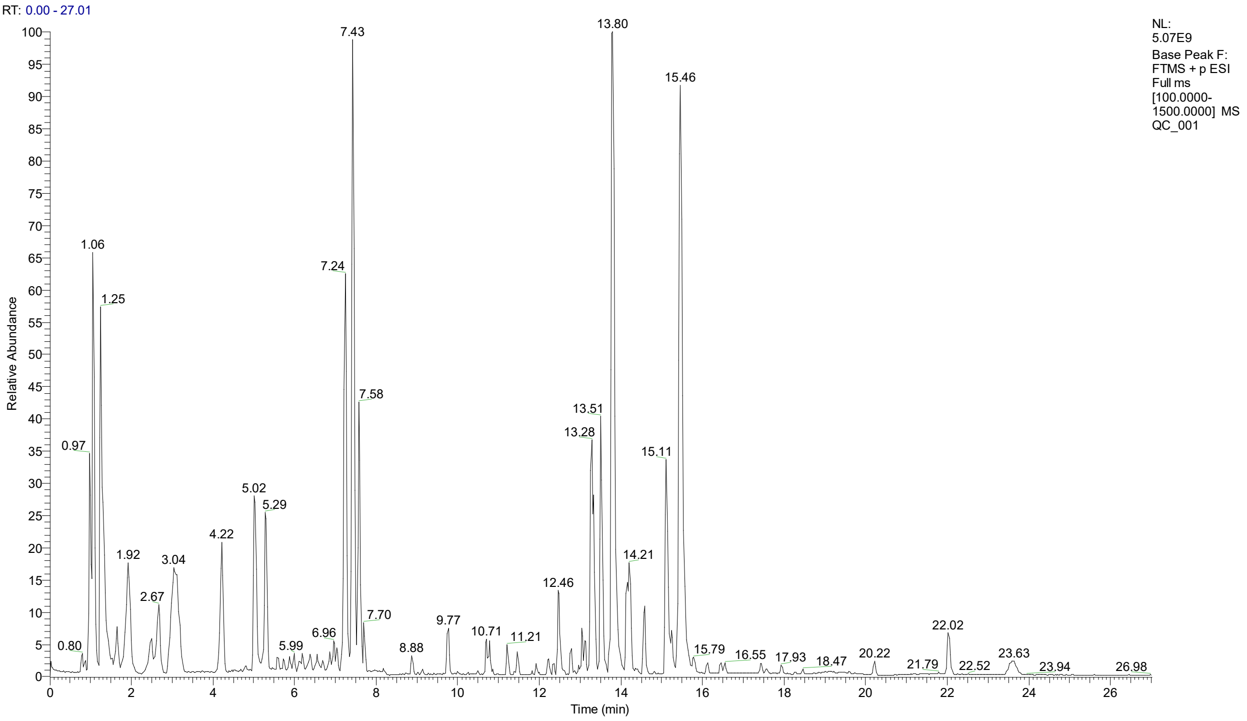

Figure S3: The raw chromatography of plasma metabolome (This chromatography is derived from the positive and negative ion co-sweeping.)
